# A retrospective cross-sectional study for predicting 72-h mortality in patients with serum aspartate aminotransferase levels ≥ 3000 U/L

**DOI:** 10.1038/s41598-020-79435-3

**Published:** 2021-01-12

**Authors:** Kai Saito, Hitoshi Sugawara, Tamami Watanabe, Akira Ishii, Takahiko Fukuchi

**Affiliations:** 1 Nara Medical University, 840 Shijo-Cho, Kashihara-shi, Nara Japan; 2 Division of General Medicine, Department of Comprehensive Medicine 1, Saitama Medical Center, Jichi Medical University, 1-847, Amanuma-cho, Omiya-ku, Saitama-shi, Saitama 330-8503 Japan

**Keywords:** Biomarkers, Cardiology, Diseases, Medical research, Pathogenesis, Risk factors, Chemistry

## Abstract

Risk factors associated with 72-h mortality in patients with extremely high serum aspartate aminotransferase levels (AST; ≥ 3000 U/L) are unknown. This single-centre, retrospective, case-controlled, cross-sectional study obtained data from medical records of adult patients treated at Saitama Medical Center, Japan, from 2005 to 2019. We conducted a multivariate logistic after adjusting for age, sex, height, weight, body mass index, Brinkman Index, vital signs, biochemical values, updated Charlson Comorbidity Index (CCI) score, CCI components, and underlying causes. A logistic regression model with selected validity risks and higher C-statistic for predicting 72-h mortality was established. During the 15-year period, 428 patients (133 non-survivors and 295 survivors [cases and controls by survival < 72 and ≥ 72 h, respectively]) with AST levels ≥ 3000 U/L were identified. The 72-h mortality rate was 133/428 (31.1%). The model used for predicting 72-h mortality through the assessment of alkaline phosphatase, creatine kinase, serum sodium, potassium, and phosphorus levels had a C-statistic value of 0.852 (sensitivity and specificity, 76.6%). The main independent risk factors associated with 72-h mortality among patients with AST levels ≥ 3000 U/L included higher serum values of alkaline phosphatase, creatine kinase, serum sodium, potassium, and phosphorus.

## Introduction

Clinical laboratory data are accumulated daily at each medical institution, and clinical data reuse or secondary use is essential for improved healthcare management^1^. However, laboratory databases have seldom been fully reused to improve the overall quality of medical care.

Critical values represent a pathophysiological state so different from normal functioning that it becomes life-threatening if no prompt action is taken. The values for which an urgent corrective action is needed include serum potassium (K) levels ≤ 2.6 mmol/L^2^. Extreme outlier values are statistically expressed as those below the 0.5–1.0th percentiles or above the 99.0–99.5th percentiles^3^. However, physicians who treat patients with extreme outlier values may not know which of these laboratory parameters are critical values.

Considering the importance of prognosis prediction, several models have been established. The Acute Physiology and Chronic Health Evaluation (APACHE) IV model, utilized for predicting hospital mortality among critically ill adults^4^, determines the association between acute changes in a patient's physiologic balance and the short-term mortality risk.

Serum aspartate aminotransferase (AST) is a transaminase enzyme that catalyses the conversion of aspartate and α-ketoglutarate to oxaloacetate and glutamate^5^. AST is present in cells across all organs except the bone, with the highest levels found in the liver, heart, and skeletal muscles^6^. High AST levels may be caused by considerable tissue damage and have low specificity for any single disease. However, marked increases in AST levels are useful for prognostic and clinical prediction. Johnson et al., who first reported the prognosis of patients with extreme AST level elevations (≥ 3000 U/L), concluded that serum AST concentrations of 3000 U/L or higher occurred in approximately 2 per 1000 admissions, and that extreme AST elevations were most often attributable to hypoxic hepatitis^7^. Other reported aetiologies for serum AST levels ≥ 3000 U/L include drug/toxin hepatitis (acetaminophen, ciprofloxacin, phenytoin, isoniazid, and cocaine), liver trauma, viral hepatitis, heart failure, hepatic metastases, and rhabdomyolysis^7^. Patients with extreme AST levels due to hypoxic hepatitis had a 75% in-hospital mortality rate^7^. The APACHE IV elements do not include AST levels^4^, although elevated AST levels and the AST/alanine aminotransferase (ALT) ratio has been found to be useful for determining the potential mortality risk of individuals with solid tumours^8^ and cardiovascular diseases^9,10^ as well as in ascertaining all-cause mortality^8^. However, the very early prognosis of patients with extremely high outlier values of AST remains unknown.

This retrospective study aimed to investigate the association between very early mortality risk, defined as death within 72 h^11,12^ following testing, of patients exhibiting extremely high outlier values of AST, and validity risks. In clinical practice, predicting 72-h mortality is extremely important for estimating and improving patient outcome in both primary and critical care settings. Siregar et al. described a 72-h mortality prediction model for patients with diabetic ketoacidosis in Indonesia^11^. Another study involving a Singaporean cohort validated 72-h mortality among patients with sepsis by using the emergency department sepsis score^12^.

## Results

### Participant demographics

**Figure 1:**
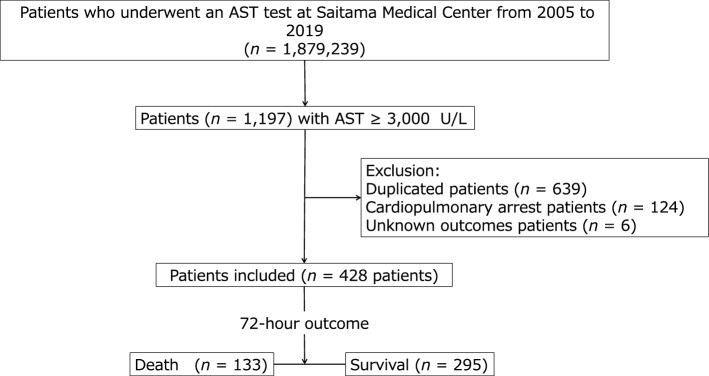
Flow diagram of patient selection. Among the 1,879,239 patients who underwent testing at our medical centre’s clinical laboratory, 1197 had extremely high outlier values of serum aspartate aminotransferase (AST; ≥ 3000 U/L) during a 15-year period, from 2005 to 2019. After applying the exclusion criteria, 124 patients with cardiopulmonary arrest upon arrival, 6 with unknown outcomes, and 639 metachronous duplicates of the same patient were excluded. Finally, 428 patients, including 133 non-survivors (died within 72 h) and 295 survivors (survived beyond 72 h) were enrolled in this study.

This study enrolled 428 patients (Fig. 1), among whom 133 were non-survivors (death within 72 h) and 295 were survivors (control), with a 72-h mortality rate of 31.1%. The sample size included in this study allowed for a power of 80% with an alpha error of 5%. Patient characteristics are presented in Tables 1 and 2. Non-survivors had significantly higher age, respiratory rate (RR), and levels of total bilirubin (T-Bili), direct bilirubin (D-Bili), AST, lactate dehydrogenase (LDH), ALP, creatine kinase (CK), amylase, sodium (Na), potassium (K), and phosphorus (P), whereas survivors had significantly higher levels of systolic blood pressure (SBP), diastolic blood pressure (DBP), body temperature (BT) and levels of total protein (TP), albumin (ALB), and calcium (Ca). No group differences in sex, height, weight, BMI, the Brinkman Index, pulse rate, random plasma glucose (RPG), ALT, γ-glutamyl transpeptidase (γ-GTP), C-reactive protein (CRP), chloride (Cl), magnesium (Mg), blood urea nitrogen (BUN), creatinine (Cre), uric acid (UA), total cholesterol (T-C), low-density lipoprotein cholesterol (LDL-C), high-density lipoprotein cholesterol (HDL-C), and triglyceride (TG) were found (Table 1).

**Table 1:** Characteristics, demographics, vital signs, laboratory test values, and updated Charlson Comorbidity Index score of patients included in this study.

	72-h outcome							*P* value
	Death (n = 133)			Survival (n = 295)				
	Median	2.5–97.5 percentile	Number (%)	Median		2.5–97.5 percentile	Number (%)	
Age (years)	71.0	25.5–89.0	133	68.0		21.0–87.1	295	0.0221
Sex (male)			93 (69.9)				196 (66.1)	0.4353
Height (cm)	161.0	137.8–181.7	121	162.0		142.4–180.0	287	0.3029
Weight (kg)	57.5	36.1–98.0	126	57.0		33.0–92.2	293	0.3665
Body mass index (kg/m^2^)	22.1	15.7–34.6	121	22.1		15.2–33.2	287	0.1809
Brinkman index	150.0	0.0–2505.0	96	2.5		0.0–1515.0	214	0.1824
**Vital signs**								
Systolic blood pressure (mmHg)	97.0	49.7–150.4	126	106.0		70.3–163.8	290	0.0000
Diastolic blood pressure (mmHg)	53.0	27.6–116.3	125	60.0		31.3–100.7	286	0.0003
Pulse rate (beats/min)	95.0	48.6–153.9	123	92.0		53.7–141.0	287	0.1215
Respiratory rate (breath/min)	25.0	11.1–45.5	115	20.0		10.8–36.7	263	0.0000
Body temperature (°C)	36.50	33.00–41.05	120	36.80		35.00–39.10	277	0.0310
**Biochemical examinations**								
Random plasma glucose (mg/dL)	122.0	31.7–332.4	51	131.0		40.5–412.0	160	0.5010
Total protein (g/dL)	4.80	2.70–7.20	100	5.40		3.40–7.65	239	0.0000
Albumin (g/dL)	2.30	0.90–3.80	101	2.80		1.55–4.35	239	0.0001
Total bilirubin (mg/dL)	2.68	0.48–17.29	126	1.83		0.49–10.77	279	0.0005
Direct bilirubin (mg/dL)	1.82	0.31–13.39	126	1.07		0.27–6.58	279	0.0000
Aspartate transaminase (U/L)	6659.0	3155.1–23,723.6	133	4974.0		3058.6–19,001.4	295	0.0000
Alanine transaminase (U/L)	2097.0	354.0–7399.7	133	2284.0		408.4–6649.0	295	0.1530
γ-Glutamyl transferase (U/L)	70.0	14.0–713.8	99	86.0		20.0–757.5	227	0.0526
Lactate dehydrogenase (U/L)	8910.0	3095.5–38,605.0	133	5773.0		1536.2–20,696.0	288	0.0000
Alkaline phosphatase (U/L)	411.0	165.2–3690.0	107	309.0		123.6–1974.1	243	0.0000
Creatine kinase (U/L)	3325.0	102.2–142,540.0	124	1000.0		35.7–103,293.4	273	0.0000
Amylase (U/L)	263.0	41.6–2373.7	41	169.0		18.9–2040.9	94	0.0172
C-reactive protein (mg/dL)	7.17	0.29–27.21	67	6.63		0.12–24.73	167	0.0543
Sodium (mEq/L)	139.0	127.6–156.0	131	138.0		128.5–149.5	279	0.0155
Potassium (mEq/L)	5.40	3.70–7.96	131	4.60		3.50–6.56	279	0.0000
Chloride (mEq/L)	103.0	91.8–112.5	131	103.0		92.0–114.5	279	0.2836
Calcium (mg/dL)	7.50	5.70–9.47	126	7.70		5.94–9.70	277	0.0062
Phosphorus (mg/dL)	6.60	2.89–13.59	126	5.00		2.44–8.72	277	0.0000
Magnesium (mg/dL)	2.40	1.61–3.59	103	2.30		1.50–3.50	228	0.0606
Blood urea nitrogen (mg/dL)	41.0	6.8–122.7	133	40.0		10.0–96.4	295	0.9983
Creatinine (mg/dL)	2.10	0.44–5.46	133	2.04		0.555–6.51	295	0.4652
Uric acid (mg/dL)	9.35	2.22–18.37	72	8.40		3.09–17.91	177	0.3571
Total cholesterol (mg/dL)	120.5	41.6–222.0	32	126.0		61.8–221.4	99	0.4185
Low density lipoprotein cholesterol (mg/dL)	63.0	27.0–131.2	12	64.5		13.6–176.6	52	0.8973
High density lipoprotein cholesterol (mg/dL)	35.0	13.0–58.0	13	31.0		8.4–78.5	65	0.3342
Triglyceride (mg/dL)	76.0	27.3–387.7	29	69.0		19.0–691.5	90	0.7219
**Updated Charlson Comorbidity Index**								
Total score	6.0	0.0–13.0	133	6.0		0.0–12.0	295	0.1863

*P-*values were calculated using the Mann–Whitney *U* test.

**Table 2:** Patient characteristics, demographics, components of the Charlson Comorbidity Index, and underlying causes.

	72-h outcome		Total	*P* value
	Death (*N* = 133)	Survival (*N* = 295)		
	*n* (%)	*n* (%)	*n* (%)	
**Components of updated Charlson Comorbidity Index (score)**				
Congestive heart failure (2)	42 (31.6)	113 (38.6)	155 (36.2)	0.1648
Dementia (2)	4 (3.0)	7 (2.4)	11 (2.6)	0.7547
Chronic pulmonary disease (1)	6 (4.5)	19 (6.5)	25 (5.8)	0.4220
Rheumatologic disease (1)	1 (0.8)	7 (2.4)	8 (1.9)	0.4443
Mild liver disease (2)	8 (6.0)	16 (5.5)	24 (5.6)	0.8245
Hemiplegia or paraplegia (2)	4 (3.0)	9 (3.1)	13 (3.0)	1.0000
Mild to severe renal disease (1)	37 (27.8)	87 (29.5)	124 (29.0)	0.7242
Diabetes with chronic complications or history of DKA (1)	14 (10.5)	29 (9.9)	43 (10.0)	0.8418
Any malignancy, including leukaemia and lymphoma (2)	39 (29.3)	66 (22.5)	105 (24.5)	0.1314
Moderate or severe liver disease (4)	125 (94.0)	279 (94.6)	404 (94.4)	0.8056
Metastatic solid tumour (6)	5 (3.8)	11 (3.8)	16 (3.7)	0.9969
Acquired immunodeficiency syndrome/HIV infection (4)	0 (0.0)	0 (0.0)	0 (0.0)	N/A
**Underlying causes**				
Sepsis	26 (19.5)	23 (7.8)	49 (11.4)	0.0004
Pneumonia	8 (6.0)	9 (3.1)	17 (4.0)	0.1462
Infectious diseases other than sepsis and pneumonia	7 (5.3)	5 (1.7)	12 (2.8)	0.0548
Malignancy	28 (21.1)	47 (15.9)	75 (17.5)	0.1972
Hepatic disorder	6 (4.5)	35 (11.9)	41 (9.6)	0.0168
Heart failure	4 (3.0)	45 (15.3)	49 (11.4)	0.0002
Cerebrovascular disease	43 (32.3)	98 (33.2)	141 (32.9)	0.8562
Autoimmune disease	1 (0.8)	5 (1.7)	6 (1.4)	0.6707
Others	10 (7.5)	28 (9.5)	38 (8.9)	0.5067
Total	133 (100.0)	295 (100.0)	428 (100.0)	N/A

DKA, diabetic ketoacidosis; HIV, human immunodeficiency virus. *P-values* were calculated using Fisher’s exact test.

Regarding updated Charlson comorbidity index (CCI)^13^ components (Table 2), there was no significant difference between groups. However, significant intergroup differences in the underlying causes of the proportion of sepsis, hepatic disorders, and heart failure were observed (Table 2).

### Univariate logistic regression analysis

The crude (unadjusted) regression analysis revealed the following significant risk factors associated with an increase in the primary outcome: age, Brinkman Index, RR, the presence of sepsis or infectious diseases besides sepsis or pneumonia, T-Bili, D-Bili, AST, LDH, ALP, CK, amylase, Na, K, and P. Risk factors that showed a protective effect included SBP, DBP, the presence of a hepatic disorder or heart failure, TP, ALB, γ-GTP, and Ca. The K level had the highest C-statistic (0.728; Table 3).

**Table 3:** Univariate and multivariate logistic regression analyses of specific variables.

	Univariate logistic regression analysis			Multivariate logistic regression analysis			
	Crude odds ratio (95% CI)	*P* value	C-statistic	Age- and AST-adjusted odds ratio (95% CI)	*P* value	C-statistic	VIF
Age	1.015 (1.001–1.029)	0.0342	0.569	1.022 (1.007–1.037)	0.0037	0.657	1.020
Brinkman Index	1.000 (1.000–1.001)	0.0404	0.545	1.000 (1.000–1.001)	0.0745	0.706	1.014
**Vital signs**							
Systolic blood pressure (mmHg)	0.978 (0.969–0.988)	0.0000	0.636	0.980 (0.971–0.990)	0.0000	0.699	1.019
Diastolic blood pressure (mmHg)	0.985 (0.974–0.997)	0.0131	0.611	0.990 (0.978–1.002)	0.1089	0.658	1.041
Respiratory rate (breaths/min)	1.078 (1.046–1.111)	0.000	0.701	1.079 (1.047–1.112)	0.0000	0.737	1.018
**Underlying causes**							
Sepsis	2.874 (1.571–5.257)	0.0006	0.559	2.288 (1.219–4.294)	0.0100	0.685	1.037
Infectious diseases besides sepsis and pneumonia	3.222 (1.004–10.346)	0.0493	0.518	3.134 (0.939–10.456)	0.0631	0.661	1.001
Hepatic disorder	0.351 (0.144–0.856)	0.02135	0.537	0.420 (0.168–1.049)	0.0633	0.665	1.040
Heart failure	0.172 (0.061–0.490)	0.0010	0.561	0.184 (0.640–0.530)	0.0017	0.692	1.005
**Biochemical examinations**							
Total protein (g/dL)	0.637 (0.516–0.786)	0.0000	0.646	0.647 (0.536–0.831)	0.0003	0.702	1.029
Albumin (g/dL)	0.350 (0.242–0.507)	0.0000	0.692	0.358 (0.242–0.530)	0.0000	0.734	1.035
Total bilirubin (mg/dL)	1.477 (1.206–1.808)	0.0002	0.608	1.486(1.186–1.862)	0.0006	0.690	1.142
Direct bilirubin (mg/dL)	1.661(1.345–2.051)	0.0001	0.636	1.723 (1.369–2.183)	0.0000	0.709	1.143
Aspartate aminotransaminase (U/L)	1.105 (1.059–1.154)	0.0000	0.636	1.120 (1.071–1.172)	0.0000	0.657	1.020
γ-Glutamyl transferase (U/L)	0.904 (0.820–0.997)	0.0428	0.568	0.893 (0.801–0.996)	0.0421	0.691	1.101
Lactate dehydrogenase (U/L)	1.031 (1.022–1.040)	0.0000	0.715	1.048 (1.031–1.064)	0.0000	0.733	2.757
Alkaline phosphatase (U/L)	1.179 (1.094–1.270)	0.0000	0.638	1.167 (1.077–1.265)	0.0002	0.709	1.055
Creatine kinase (U/L)	1.317 (1.178–1.473)	0.0000	0.667	1.275 (1.132–1.436)	0.0001	0.700	1.101
Amylase (U/L)	1.103 (1.003–1.213)	0.0426	0.629	1.071 (0.968–1.184)	0.1827	0.685	1.060
Sodium (mEq/L)	2.296 (1.185–4.450)	0.0138	0.574	2.040 (1.035–4.021)	0.0395	0.669	1.020
Potassium (mEq/L)	10.824 (5.646–20.753)	0.0000	0.728	9.966 (5.103–19.46)	0.0000	0.762	1.028
Calcium (mg/dL)	0.729 (0.575–0.924)	0.0090	0.585	0.741 (0.578–0.949)	0.0175	0.662	1.008
Phosphorus (mg/dL)	1.785 (1.520–2.095)	0.0000	0.720	1.771 (1.501–2.089)	0.0000	0.756	1.027

CI, confidence interval; AST, aspartate aminotransferase; VIF, variance inflation factor. *P*-values were calculated using the χ^2^ test.

### Multivariate logistic regression analysis

After adjusting for age and AST, the strongest risk factor for increased risk for 72-h mortality was the K level, which also had the highest C-statistic (0.762; Table 3). Except for the Brinkman index, DBP, the presence of infectious diseases besides sepsis or pneumonia, the presence of hepatic disorders, and amylase levels, the other risk factors tested using the univariate model remained significant.

### Prediction model

Five covariates (i.e., ALP, CK, Na, K, and P) that were determined to be the most significant predictors were selected for stepwise regression analysis. The best combination of covariates that predicted the 72-h mortality risk with the highest C-statistic comprised serum ALP, CK, Na, K, and P (C-statistic: 0.852), and surpassed the performance of K alone obtained during multivariate logistic regression analysis (MLRA) (Table 4). The predictive probability (*p*) for 72-h mortality of the final prediction model was calculated using the following formula:

$$p = { 1}/ \, \left[ {{1 } + {\text{ exp }}\left( { - {19}.{98 } + \, 0.{29}0\left( {ALP^{0.2} } \right) \, + \, 0.{\text{348log }}\left( {CK} \right) \, + { 1}.{766}\left( {Na^{0.2} } \right) \, + { 1}.{568}\left( {K^{0.2} } \right) \, + \, 0.{474}\left( {P^{0.8} } \right)} \right)} \right].$$

**Table 4:** Comparing multivariate logistic regression analysis for predicting 72-h mortality.

	Exp Var	β	SE (β)	z	*P* value	VIF	Odds ratio	95%CI	n (with all Exp Vars)	AIC
**Reference model**										
0		− 4.586	0.7820						428	505.5
1	Age	0.022	0.0075	2.906	0.0037	1.020	1.022	1.007–1.037		
2	AST	0.114	0.0229	4.965	0.0000	1.020	1.120	1.071–1.172		
**Prediction model**										
0		− 19.98	3.6070						303	269.7
1	ALP	0.290	0.0601	4.824	0.0000	1.078	1.337	1.188–1.504		
2	CK	0.348	0.0796	4.365	0.0000	1.112	1.416	1.211–1.655		
3	Na	1.766	0.5617	3.145	0.0017	1.119	5.850	1.946–17.59		
4	K	1.568	0.4755	3.298	0.0010	1.522	4.796	1.889–12.18		
5	P	0.474	0.1145	4.145	0.0000	1.399	1.607	1.284–2.011		
*P* = 1/ [1 + exp (− 19.98 + 0.290*(ALP*^*0.2*^*)* + 0.348log *(CK)* + 1.766*(Na*^*0.2*^*)* + 1.568*(K*^*0.2*^*)* + 0.474*(P*^*0.8*^*)*)]										

n, number; Exp Var, explanatory variable; SE, standard error; z, z value; VIF, variance inflation factor; CI, confidence interval; AIC, Akaike's information criterion; exp, exponential function; log, logarithm, Sn, sensitivity; Sp, specificity; AST, aspartate transaminase; ALP, alkaline phosphatase; Na, sodium; K, potassium; and P, phosphorus. *P*-values were calculated using the chi-square test.

**Figure 2:**
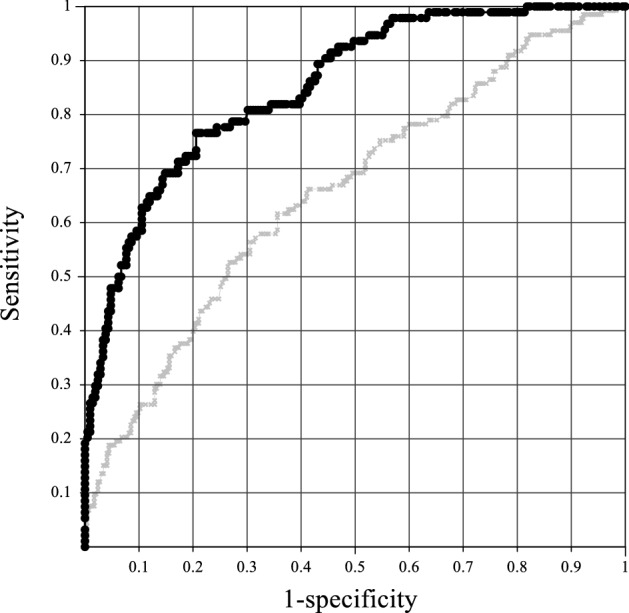
Receiver operating characteristic curve analysis of models that predicted 72-h mortality among patients with extremely high values of serum aspartate aminotransferase. Gray line indicates reference values, comprising age and aspartate aminotransferase (AST); black line indicates prediction mode, comprising alkaline phosphatase (ALP), creatine kinase (CK), sodium, pottasium, and phosphorus values. The predicted probability (*p*) for the 72-h mortality of the prediction model was calculated using the following formula: *p* = 1/ [1 + exp (-19.98 + 0.290(*ALP*^*0.2*^) + 0.348log (*CK*) + 1.766(*Na*^*0.2*^) + 1.568(*K*^*0.2*^) + 0.474(*P*^*0.8*^))].

This model had 76.6% sensitivity and specificity for predicting 72-h mortality among patients with extremely high outlier values of AST (Table 5; Fig. 2).

**Table 5:** Comparing of receiver-operator characteristic analysis of the models to evaluate the accuracy of predicted probability (p) for the 72-h mortality.

N dead	N alive	C-statistics	95%CI of C-statics	Sn (%)	Sp (%)
**Reference model**					
133	295	0.657	0.600–0.713	62.3	62.3
**Prediction model**					
94	209	0.852	0.806–0.897	76.6	76.6

N, number; CI, confidence interval; Sn, sensitivity; Sp, specificity.

## Discussion

This study revealed that an extremely high outlier value of AST (≥ 3000 U/L) was a critical value. Because the 72-h mortality rate among 133 patients with AST ≥ 3000 U/L was 31.1%. The mortality risk was associated with higher values of ALP, CK, Na, K, and P, regardless of serum AST levels and age. The reason for the association between the 72-h mortality rate among the participants with extremely high outlier values of AST and high serum ALP, CK, Na, K, and P remains unknown. The distinctive profile of underlying causes among non-survivors (i.e., significantly higher proportions of sepsis and significantly lower proportions of hepatic disease and heart failure) may be one of the reasons. However, this does not imply that patients with cardiac or liver disease have a better prognosis and, instead, may indicate that advances in treatments and interventions for these two disorders have decreased the likelihood of short-term mortality within 72 h in patients with markedly increased AST. The long-term prognosis of these patients still needs to be closely monitored.

This study showed a significant difference in AST levels between survivors and non-survivors. One study showed that, among patients with severe hypoxic hepatitis, indicated by mean AST levels of 4896 U/L, low serum albumin levels predicted early mortality^14^. Another study reported a strong relationship between aminotransferase concentrations, including AST, and mortality from liver disease^15^. Koehler et al. reported that AST was positively associated with all-cause mortality among the elderly population^16^, whereas a higher AST level (> 20 U/L) was reported to be incrementally associated with higher mortality among patients receiving maintenance haemodialysis^17^. However, the previously mentioned studies did not include extremely high outlier values for AST, and the follow-up period in such studies was more than 1 year. Srivatsan et al. reported that the urinary albumin–creatinine ratio, ALT, AST, and prothrombin time/international normalized ratio were significantly correlated with APACHE II scores and mortality among patients with systemic inflammatory response syndrome^18^, suggesting that extremely high outlier values of AST are associated with short- or long-term mortality.

The serum ALP level was associated with 72-h mortality among patients with extremely high outlier values of AST. Increased ALP levels could be a risk factor for mortality among patients with acute coronary syndromes^19,20^. Furthermore, ALP was related to long-term mortality in an elderly population^16^, suggesting that high ALP levels may affect short-term prognosis.

The median serum sodium value among the non-survivors included in this study was 139.0 mEq/L, which may be considered a normal value; however, a 1.0 mEq/L increase in the serum sodium concentration increased the odds ratio for 72-h mortality by 2.30. Hypernatremia > 150 mEq/L is uncommon^21,22^ and it is an independent risk factor for inpatient mortality and poor outcomes among patients with acute subarachnoid haemorrhage^21^ and those admitted to the intensive care unit with comorbid organ dysfunction^23^. Persistent hypernatremia is a strong risk factor for in-hospital mortality among patients with various diseases^22^. These reports support the association between higher sodium levels and poor outcomes among patients with extremely high outlier values of AST.

The non-survivors included here had a median serum phosphorus value of 6.60 mg/dL. A serum phosphorus level greater than 5.50 mg/dL was associated with a further increase in the risk of cardiovascular and all-cause mortality among patients with mild or moderate renal dysfunction^24^. In contrast, survivors in this study had a median phosphorus value of 5.00 mg/dL, which was significantly lower than that for non-survivors. This difference might have affected the early prognosis. Dhingra et al. reported an association between all-cause mortality within 45 months among patients with chronic kidney disease and hyperphosphataemia^25^. High serum phosphorus levels are associated with the risk of cardiovascular disease, which leads to high mortality rates^17^. Naffa et al. reported that high phosphorus levels affected the mortality rate of patients with pneumonia or type 2 diabetes mellitus^26^. Another study conducted at our hospital that included patients with extremely high CRP levels suggested that serum phosphorus might serve as a good predictor of 72-h mortality^27^. These findings suggest that phosphorus is associated with various types of diseases and might be an important variable for predicting short-term prognosis.

Both the increase in RPG, T-Bili, AST, ALT, ALP, BUN, Cre, Na, K, Mg, P, and lactate levels as well as the decrease in haemoglobin, platelets, ALB, Ca, and bicarbonate levels were reported as end-of-life laboratory values^28,29^. Regarding other risk variables that were identified by the MLRA, K and CK are well-documented mortality risk factors. Hyperkalaemia prior to death can be explained by renal failure, apoptotic release from cells in multi-organ failure, or increased intravascular haemolysis. The elevation of serum CK levels is known to be commonly caused by hypoxaemia in muscle circulatory failure and leakage from damaged cells in skeletal and cardiac muscles. High CK and hyperkalaemia are linked to each other and are associated with renal failure.

Patients with a marked increase in aminotransferase levels (> 10 times the upper reference limit) typically have acute viral hepatitis or toxic or ischemic liver injury^6^. However, a previous study showed that extremely high outlier values of AST were not always attributable to ischemic hepatitis^7^. In this study, the major underlying causes of high AST value with a prevalence of 10% or more included sepsis, malignancy, heart failure, cerebrovascular disease such as aortic aneurysm or dissection, myocardial infarction, acute arterial obstruction, and stroke. As hypoxic hepatitis and ischemic hepatitis are pathological rather than clinical terms, it remains unclear as to what proportion of patients with malignant disease had hypoxic hepatitis among the non-survivors.

## Limitations

Several limitations of this study need to be mentioned. First, considering that this was a single-centre retrospective study conducted at Saitama Medical Center in Japan, the findings presented herein may not be generalisable to other patients. Second, given that patients with AST levels equal to greater than 3000 U/L were selected, risk factors for 72-h mortality may be different among patients with elevated AST levels that are lower than the set threshold.

## Conclusions and future research

This study found that an extremely high outlier value of AST (≥ 3000 U/L) could be a critical value that resulted in higher mortality rates and was associated with higher serum values of ALP, CK, Na, K, and P, regardless of AST values and age. Therefore, physicians treating patients with AST levels ≥ 3000 U/L can easily estimate the probability of 72-h mortality by focusing on ALP, CK, Na, K, and P levels to anticipate disease conditions, explain the prognosis to the patient and their family, and make appropriate decisions on subsequent treatments.

This study could not determine why 72-h mortality among those with extremely high outlier values of AST was associated with high serum ALP, CK, Na, K, and P levels. Moreover, the formula presented herein remains untested. Thus, these preliminary findings warrant testing in prospective studies to validate the performance of the mortality prediction model among patients with extremely high outlier values of AST to identify the mechanisms whereby higher ALP, CK, Na, and P levels lead to increased 72-h mortality, and to prevent mortality associated with extremely high outlier values of AST.

We believe that 72-h mortality prediction among patients with extremely high outlier values of AST can help physicians make prompt decisions and provide therapeutic and management options to patients and their families, which should, in turn, improve the quality of initial medical management.

## Methods

The study protocol was designed in accordance with the tenets of the Declaration of Helsinki^30^. The Institutional Clinical Research Ethics Review Board of Saitama Medical Center, Jichi Medical University, Saitama, Japan approved this study (Clinical #10-79 and #S20-025) and waived the requirement to obtain informed consent because of the retrospective design.

### Study design and patient selection

This retrospective, single-centre, case-controlled cross-sectional study enrolled participants were selected through a chart review of the medical records of Saitama Medical Centre, Japan. We identified a cohort of 1,879,239 patients aged > 18 years who underwent blood biochemical examinations, including AST, at the hospital’s clinical laboratory within a 15-year period from 2005 to 2019. Among the identified patients, 1197 had extremely high outlier values of AST, defined as levels more than 100 times the upper limit of normal (reference range: 13–30 U/L), that is, ≥ 3000 U/L (occurrence rate ~ 0.0637%), as previously reported^7^. Patients who were metachronous duplicates of the same patient (only one highest AST value from each patient was considered), those who experienced cardiopulmonary arrest on arrival, and those with unknown outcomes were excluded. After applying the exclusion criteria, a sample of 428 patients was selected for further analysis. A flow chart of the cohort selection process is presented in Fig. 1.

The primary outcome was defined as 72-h mortality^11,12^ following the AST test, regardless of symptom onset, hospitalisation, or setting (i.e., emergency or outpatient). Cases were defined as patients with extremely high outlier values of AST who died in the hospital during the first 72 h after the test, whereas controls were defined as patients with extremely high outlier values of AST who survived beyond 72 h.

The following risk factors were tested for their association with 72-h mortality: age, sex, height, BW, BMI, number of cigarettes smoked (Brinkman Index), vital signs upon examination (e.g., SBP, DBP, pulse rate [PR], RR, and BT), laboratory test values (e.g., RPG, total protein [TP], ALB, T-Bili, D-Bili, AST, ALT, γ-GTP, LDH, ALP, CK, amylase, CRP, Na, K, Cl, Ca, P, Mg, BUN, Cre, UA, T-C, LDL-C, HDL-C, and TG), comorbidities defined according to the updated CCI^13^, and underlying causes. The vital signs were recorded either at the time or as close to the time of blood sample collection as possible.

### Statistical analysis

#### Sample size

From a preliminary analysis, we obtained a dead survival ratio of 30:70 (mortality rate of 30%) among patients with AST levels ≥ 3000 U/L. We calculated the required sample size using G*Power software^31^. In order to compute the required sample size given α, power, and effect size, we input the parameters as follows: the platforms of Test family, Exact; Statistical test, Proportions: Inequality, two independent groups (Fisher’s exact test); and type of power analysis, A priori. Assuming the need to test the utility of a binary risk variable by assessing the proportions of the two groups, the sample size that was required to detect a difference of 0.15 in proportion was calculated as 236 (71 for the dead group vs. 165 for the surviving group) by setting tails of two, a power of 80%, an alpha error of 5%, and an allocation ratio of 0.429. Thus, we expanded the actual data size to 428 (with expected data sizes of 133 vs. 295 for the non-survivor and survivor groups, respectively) to ensure attainment of a higher power.

### Descriptive statistics

All continuous data were evaluated assuming normality. All variables are presented as median and 2.5–97.5th percentiles. However, only median values are presented where the amount of data was inadequate to calculate the 2.5–97.5th percentile. Differences between groups were determined using Fisher’s exact test for nominal variables and the Mann–Whitney *U* test for continuous variables.

### Association analysis

Our primary focus was to determine multiple risk factors associated with 72-h mortality. For continuous variables with a skewed distribution, the distribution of values for each risk factor was made approximately Gaussian by power transformation using the Box–Cox formula^32^ for regression analyses.

$$X= \frac{{x}^{\lambda }-1}{\lambda } \cdots \left(\lambda \ne 0.0\right); X=log(x)\cdots \left(\lambda =0.0\right)$$

where *x* and *X* are the test results before and after the transformation, and λ is the power.

The power used for the major laboratory tests was λ = 0.0 (log-transformation) for BW, CK, T-C, LDL-C, and TG; λ = 0.2 for T-Bili, D-Bili, AST, ALT, ALP, γ-GTP, amylase, CRP, and Na; λ = 0.4 for LDH and RPG; λ = 0.6 for HDL-C; and λ = 0.6 for BUN, Cre, Cl, and P.

First, potential risk factors for 72-h mortality were determined using univariate logistic regression analysis. Odds ratios (ORs) and 95% confidence intervals (CIs) were estimated without adjustment (crude OR in univariate regression analysis). C-statistics for covariates with significant crude ORs were determined using receiver operating characteristic (ROC) analysis.

The MLRA was subsequently performed using the listwise deletion, which is a method for handling missing data. In this method, an entire record was excluded from the analysis if any single value was missing. ORs and 95% CI were then determined after adjusting for age and AST level for all covariates that were significant during univariate logistic regression analysis and the C-statistic using ROC analysis. Clinically important risk factors determined to be significant during MLRA (including association analysis for age and AST level) were included in the final prediction model.

The final prediction model was estimated using MLRA with a stepwise selection method, which was used to obtain an optimal combination of risk variables while simultaneously removing those deemed unimportant. In the final prediction model, the logit function reflected the probability of death, defined as 72-h mortality.

The following statistical variables were estimated during MLRA and ROC analyses: intercept, regression coefficient, standard error, z value, *P*-value, adjusted ORs, 95% CIs, variance inflation factor (VIF), C-statistic, sensitivity, and specificity. A value of *P* < 0.05 was considered statistically significant. The C-statistic was defined as the area under the ROC curve. In addition, multiple collinearities between covariates were assessed using the VIF. Multiple collinearities were defined as positive when the VIF was ≥ 5.

### Statistical software

The statistical package for the StatFlex software version 7.0.11 (Artech Co. Ltd., Osaka, Japan) was used for data analysis, while G*Power version 3.1.9.4 (Heinrich Heine University Düsseldorf, Düsseldorf, Germany)^31^ was used for sample size calculation.

## Data Availability

The datasets generated and/or analysed during the current study are available from the corresponding author upon reasonable request.
